# Investigating the mental health status of infertile women

**DOI:** 10.18502/ijrm.v17i4.4556

**Published:** 2019-06-13

##  Dear Editor

The stressful experience of infertility is associated with a wide range of psychological damage (1), so infertility affects people's mental health and all aspects of an individual's life (2). Since women in the family are considered to be the main pillars of the community and they are also more vulnerable to illnesses, therefore consideration of their health is also very important (3). The objective of the current letter is investigating the mental health status of infertile women and its related factors as predictors of mental health in infertile women.

This is a descriptive study conducted on 100 infertile women referred to the infertility treatment centers in Mazandaran province, North of Iran. The General Health Questionnaire was provided to the infertile women. The questionnaire is a self-reporting questionnaire that is used clinically to track those who are prepared for mental illness (4).

Based on the findings, Total Scale of General Health Questionnaire was 33.18 ± 10.27 that was according to the cut-off point of 22, 85% of the infertile women in this study are in the disorder condition. Also, in terms of subscales: physical complaints was 8.65 ± 3.97, anxiety and insomnia was 8.69 ± 5.58, disruption of social function was 12.73 ± 3.34, and depression was 3.10 ± 3.79. The most common disorder was related to social disorder subscale and the least common subscale was related to the depression disorder. The subscales of physical symptoms and sleep disturbance and anxiety are ranked almost at one level, and they are classified between the two subscales that were already mentioned.

Therefore, based on our findings and the level of women's mental health, there is a need for a psychologist or midwifery counselor in the infertility treatment centers to improve the mental health of women. In addition, since the mental disease may also affect the outcome of the treatment, attention to the mental health of infertile women is really importance.

**Zeinab Hamzehgardeshi${}^{1,2,3}$ Ph.D., Fereshteh Yazdani${}^{4}$ M.Sc., Forouzan Elyasi${}^{5}$ M.D., Mahmood Moosazadeh${}^{6}$ Ph.D., Sepideh Peyvandi${}^{7}$ M.D., Keshvar Samadaee${}^{8}$ M.Sc., Maryam Shahidi${}^{9,10}$ Ph.D.**

^1^Sexual and Reproductive Health Research Center, Mazandaran University of Medical Sciences, Sari, Iran.

^2^Department of Reproductive Health and Midwifery, School of Nursing and Midwifery, Mazandaran University of Medical Sciences, Sari, Iran.

^3^Traditional and Complementary Medicine Research Center, Mazandaran University of Medical Sciences, Sari, Iran.

^4^Student Research Committee, School of Nursing and Midwifery, Shahid Beheshti University of Medical Sciences, Tehran, Iran.

^5^Department of Psychiatry, Sexual and reproductive health research center, Psychiatry and Behavioral Sciences Research Center, Addiction Institute, School of Medicine, Mazandaran University of Medical Sciences, Sari, Iran.

^6^Health Science Research Center, Addiction Institute, Mazandaran University of Medical Sciences, Sari, Iran.

^7^IVF Ward, Mazandaran University of Medical Sciences, Sari, Iran.

^8^Department of Reproductive Health and Midwifery, Tehran Nursing and Midwifery Faculty, Tehran university of medical science, Tehran, Iran.

^9^Department of Medical Physics, Mazandaran Medical University, Mazandaran, Iran.

^10^Hazrat_e Maryam Fertility Center, Sari, Iran.
